# The Effects of Age on Inflammatory and Coagulation-Fibrinolysis Response in Patients Hospitalized for Pneumonia

**DOI:** 10.1371/journal.pone.0013852

**Published:** 2010-11-04

**Authors:** Sachin Kale, Sachin Yende, Lan Kong, Amy Perkins, John A. Kellum, Anne B. Newman, Abbe N. Vallejo, Derek C. Angus, for the GenIMS Investigators

**Affiliations:** 1 The Clinical Research, Investigation, and Systems Modeling of Acute Illness (CRISMA) Laboratory, University of Pittsburgh, Pittsburgh, Pennsylvania, United States of America; 2 Department of Critical Care Medicine, University of Pittsburgh, Pittsburgh, Pennsylvania, United States of America; 3 Department of Biostatistics, Graduate School of Public Health, University of Pittsburgh, Pittsburgh, Pennsylvania, United States of America; 4 Department of Epidemiology, Graduate School of Public Health, University of Pittsburgh, Pittsburgh, Pennsylvania, United States of America; 5 Department of Pediatrics, Children's Hospital of Pittsburgh, University of Pittsburgh, Pittsburgh, Pennsylvania, United States of America; University of California Los Angeles, United States of America

## Abstract

**Objective:**

To determine whether inflammatory and hemostasis response in patients hospitalized for pneumonia varies by age and whether these differences explain higher mortality in the elderly.

**Methods:**

In an observational cohort of subjects with community-acquired pneumonia (CAP) recruited from emergency departments (ED) in 28 hospitals, we divided subjects into 5 age groups (<50, 51–64, 65–74, 75–84, and ≥85). We measured circulating levels of inflammatory (TNF, IL-6, and IL-10), hemostasis (D-dimer, Factor IX, thrombin-antithrombin complex, antithrombin and plasminogen-activator inhibitor-1), and cell-surface markers (TLR-2, TLR-4, and HLA-DR) during the first week of hospitalization and at discharge and compared 90-day mortality. We used logistic regression to compare odds ratios (OR) for 90-day mortality between age groups, adjusting for differences in pre-infection factors alone and then additionally adjusting for immune markers.

**Results:**

Of 2,183 subjects, 495, 444, 403, 583, and 258 subjects were <50, 51–64, 65–74, 75–84, and ≥85 years of age, respectively. Large age-related differences were observed in 90-day mortality (0.82% vs. 3.2% vs. 6.4% vs. 12.8% vs. 13.6%, p<0.01). No age-related differences in inflammatory and cell surface markers occurred during the first week. Older subjects had higher pro-coagulant markers on ED presentation and over first week (p≤0.03), but these differences were modest (1.0–1.7-fold differences). Odds of death for older adults changed minimally in models incorporating differences in hemostasis and inflammatory markers (for subjects ≥85 compared to those <50, OR = 4.36, when adjusted for pre-infection factors and OR = 3.49 when additionally adjusted for hemostasis markers). At discharge, despite clinical recovery as evidenced by normal vital signs in >85% subjects, older subjects had modestly increased hemostasis markers and IL-6 levels (p<0.01).

**Conclusions:**

Modest age-related increases in coagulation response occur during hospitalization for CAP; however these differences do not explain the large differences in mortality. Despite clinical recovery, immune resolution may be delayed in older adults at discharge.

## Introduction

The incidence of severe sepsis and subsequent mortality among individuals with community-acquired pneumonia (CAP) rises sharply after the age of 65 [1], [2]. For example, older individuals hospitalized with CAP have a 10-fold increased risk of severe sepsis and a 3-fold increase in 90-day mortality compared to younger adults [3], [4]. Older individuals who appear to have clinically recovered after pneumonia hospitalization also have up to a 4-fold higher mortality 1 year after hospital discharge [5], [6]. The factors underlying this phenomenon remain unclear and are not fully explained by higher pre-infection chronic disease burden or illness severity among the elderly [7], [8].

Older age has been widely characterized as a “pro-inflammatory” state [9], [10]. Chronic elevation of inflammatory and hemostasis markers has been implicated in many age-related chronic conditions, ranging from frailty syndrome to heart failure [11], [12]. Animal and human experimental models of infection also suggest that older subjects have higher circulating levels of inflammatory and coagulation-fibrinolysis response. For instance, following cecal-ligation and puncture, old mice have a 7-fold increase in circulating levels of tumor necrosis factor (TNF) and interleukin (IL)-6, and these increases are associated with higher mortality [13]. Similarly, it has been shown that old mice with bacteremia have 3-fold higher levels of hemostasis markers than young mice, which may contribute to age-related increase in endotoxin-induced thrombosis [14], [15]. Whether these differences in immune response are observed in older adults hospitalized with infection and explain age-related differences in outcome is unclear. Understanding these differences in inflammatory and coagulation-fibrinolysis response is important to design better therapeutic interventions in the elderly.

We analyzed circulating inflammatory and hemostasis markers in a large cohort of patients with CAP. We recruited patients on their arrival to the Emergency Department (ED) to compare circulating immune markers before the initiation of antibiotics and other therapeutic interventions and during the first week of hospitalization. We also examined these markers at hospital discharge, when patients appeared to have clinically recovered from infection, to compare age-related differences in resolution of immune response. We hypothesized that age-related increases in circulating inflammatory and coagulation-fibrinolysis response would occur throughout the first week and at hospital discharge, and that these differences would explain higher 90-day mortality in the elderly.

## Methods

### Ethics Statement

The Institutional Review Boards at the following hospitals approved the study: Pennsylvania: Allegheny General Hospital, Jefferson Hospital/SHHS, Mercy Hospital, St. Clair Memorial Hospital, St. Francis Medical Center, Sewickley Valley Hospital, University of Pittsburgh Medical Center (UPMC) Braddock, UPMC Horizon, UPMC Lee, UPMC McKeesport, UPMC Passavant, UPMC Presbyterian, UMPC Shadyside, UPMC Southside, UPMC St. Margaret, West Penn Hospital; Connecticut: Bridgeport Hospital, Hartford Hospital, Milford Hospital, New Britain General Hospital, Norwalk Hospital, Yale-New Haven Hospital; Tennessee: Methodist Health Care (single IRB approval for three Methodist University sites); Michigan: Henry Ford Health System, Detroit Receiving/Sinai-Grace, Wayne State. Written, informed consent was obtained from all participants or by proxy.

### Subjects and design

We analyzed data from subjects enrolled in the Genetic and Inflammatory Markers of Sepsis (GenIMS) study, a prospective multicenter observational study [16]. GenIMS recruited subjects from the emergency departments (ED) of 28 academic and community hospitals in southwestern Pennsylvania, Connecticut, southern Michigan, and western Tennessee. We enrolled adults 18 years or older who presented with CAP, as diagnosed by the criteria of Fine et al [17]. Exclusion criteria included transfer from another hospital, hospital discharge within the previous 10 days, an episode of pneumonia within the past 30 days, chronic mechanical ventilation dependency, cystic fibrosis, active pulmonary tuberculosis, positive HIV antibody titer, alcoholism with evidence of end-organ damage, admission for palliative care, incarceration, and pregnancy. We enrolled 2320 subjects and subsequently excluded 134 (6%) subjects for whom their treating physicians ruled out CAP as their primary diagnosis, thus restricting the analyses to 2183 subjects.

### Clinical characteristics

We divided the subjects into five age groups: <50 years, 50–64 years, 65–74 years, 75–84 years, and ≥85 years, which are accepted geriatric age groups and have been used elsewhere in the literature [18], [19]. We gathered baseline clinical characteristics by structured patient or proxy interviews, bedside assessment by study nurses, and structured medical record reviews. We assessed comorbidity using the Charlson comorbidity score [20]. Severity of illness upon presentation was assessed using the Acute Physiology and Chronic Health Evaluation III (APACHE III) score [21] and the Pneumonia Severity Index (PSI) [17]. We also assessed the acute physiology component of the APACHE III score to compare physiologic derangements across different age groups because age is not included in the calculation. We defined severe sepsis as pneumonia plus acute organ dysfunction following the 2001 International Consensus Criteria [22]. We defined acute organ dysfunction as a new Sepsis-related Organ Failure Assessment (SOFA) score of 3 or higher in any of 6 organ systems, based on the international Sepsis Occurrence in the Acutely ill Patient (SOAP) study [23]. We used blood and sputum cultures to determine microbiologic etiology. Lower respiratory tract secretions by bronchoscopy and pleural fluid cultures were obtained in a very small subset and did not yield additional information in our cohort. Strict criteria to define sputum quality and to exclude skin contaminants in blood cultures based on consensus guidelines were used [24]. Two reviewers reviewed all microbiology results and independently assigned etiology. We resolved any discrepancies by consensus and a third reviewer. We assessed vital signs at hospital discharge to assess whether subjects met criteria for clinical stability at hospital discharge, as described by Halm et al [25].

Our primary clinical outcome was 90-day mortality. We also compared 1-year mortality from hospital discharge in those who survived the hospitalization because age-related differences in immune response resolution, as evidenced by higher circulating immune marker levels at hospital discharge, have been associated with long-term mortality after discharge [26]. Mortality during hospitalization and after hospital discharge was ascertained by study nurses and the National Death Index search, respectively [27].

### Laboratory procedures

We assessed age-related differences in the immune response to infection during hospitalization by comparing changes in circulating concentrations of inflammatory (TNF, IL-6, IL-10), coagulation (Factor IX, thrombin-antithrombin complexes [TAT], antithrombin [AT]), and fibrinolysis (plasminogen activator inhibitor [PAI]-1 and D-dimer) systems. We assessed cell surface marker expression on monocytes and granulocytes for HLA-DR and toll-like receptor (TLR)-2 and TLR-4.

Day 1 blood samples were generally drawn immediately following enrollment in the ED. Samples were not obtained in the ED from subjects presenting after 11 PM, or on weekends and holidays for logistic reasons. Subsequent blood samples were collected at 8 AM daily for the first week and weekly thereafter until hospital discharge or day 28. Each blood sample was drawn into pyrogen-free vials containing heparin. Within one hour plasma was separated by centrifugation and divided into four 1.5 ml tubes. Plasma was then frozen at −80°C and batched and shipped on dry ice to our central laboratory.

Due to exclusion of subjects who did not have samples drawn in the ED, we analyzed Day 1 inflammatory markers in 1410 subjects. Inflammatory marker concentrations were measured using an automated chemiluminescent immunoassay analyzer (IMMULITE, Diagnostic Products Corp., Los Angeles, CA), thawing samples only once before assay. The minimal detectable levels for TNF, IL-6 and IL-10 were 4 pg/ml, 2 pg/ml and 5 pg/ml, respectively.

Day 1 hemostasis marker concentrations were analyzed in a random subset of 725 subjects by a commercial laboratory (Esoterix, Agoura Hills, CA). Specific methods and kits used were: D-dimer, latex immunoassay (Diagnostica Stago, Parsipany, NJ, USA); PAI-1, bioimmunoassay (Biopool Chromolize; Biopool International, Ventura, CA, USA); antithrombin, chromogenic (BioMerieux, Rhône-Alpes, France); Factor IX, clot (BioMerieux); and TAT, enzyme-linked immunosorbent assay (Behring, King of Prussia, PA, USA). Abnormal values were defined by the clinical laboratory or by manufacturer's assay. These abnormalities included: D-dimer >256 ng/ml, PAI-1 activity >31 IU/ml, antithrombin activity <70%, Factor IX activity <60%, and TAT >5.0 ng/ml.

Cell-surface markers were analyzed on ED presentation and on the third and seventh day of hospitalization in a subset of 523 subjects enrolled in hospitals located within 60 miles of the University of Pittsburgh because samples for cell-surface markers require analysis within 48 hours. For cell-surface marker analyses, we obtained fluorochrome- or biotin-conjugated antibodies from eBioScience (San Diego, CA, USA) for TLR-2, TLR-4, and HLA-DR. Antibodies were incubated with 100µl of whole blood for 15min at 4°C. Red blood cells were lysed, remaining cells were washed, fixed, and stored at −4°C. Cell-surface marker data were acquired within 48 hours of fixation on a BD FACS Vantage SE flow cytometer (San Jose, CA, USA) and using BD CellQuest software. Values for cell-surface markers are reported as the mean channel fluorescence of cells positive for a given cell-surface marker.

We analyzed inflammatory and hemostasis markers prior to hospital discharge in those who were alive at hospital discharge in 1739 and 893 subjects, respectively. For most subjects samples were measured within 48 hours of hospital discharge (83%). For a minority of subjects (7%), the last available cytokine concentration measurement was more than 96 hours before discharge.

### Statistical analyses

Statistical analysis was performed using SAS software, version 9.1 (SAS Institute, Cary, NC) with statistical significance set at p<0.05. We conducted univariate comparisons of clinical characteristics, risk of severe sepsis, organ dysfunction, and mortality for subjects in the five age groups using chi-square tests or Kruskal-Wallis tests where appropriate as well as linear trend tests.

We compared differences in immune response to infection, comparing immune markers in subjects in the five age groups at Day 1, during the first week of hospital stay, and at hospital discharge. We used Tobit models to account for marker levels below detection threshold and ANOVA and linear trend tests to assess age-related differences in levels in the ED and at hospital discharge [28]. For longitudinal analyses over the first week, we used regression analysis with mixed models to account for correlation of these markers over time. We assumed a log-normal distribution for marker concentrations. P values shown are adjusted for sex, race, comorbidity, and smoking status. We assessed whether discrete inflammatory cytokine patterns (IL-6:IL-10 ratio) existed among age groups over the first week because prior studies have suggested that older adults have a preponderance of either pro-inflammatory or anti-inflammatory cytokine activation, also known as immunologic dissonance [29], [30], [31]. We previously showed that for longitudinal analysis of IL-6:IL-10 patterns, 3 group models (high, medium, and low concentrations) with a quadratic trend for each group exhibited the best fit [16]. We used these models and determined the frequency of different IL-6:IL-10 patterns for each age group [32]. We determined unadjusted and adjusted odds ratios (OR) for 90-day mortality using logistic regression models, adjusting for sex, race, smoking status, and Charlson comorbidity score. We then additionally adjusted for total burden of coagulation abnormalities measured on ED presentation because hemostasis markers differed across age groups. We assigned one point for each hemostasis abnormality and input the total score (0–5) into the model.

We conducted three sensitivity analyses. First, to assess whether age-related differences between onset of symptoms of pneumonia and the time to ED presentation confounded our results, we analyzed a subgroup of subjects who reported having arrived to the ED within a day of onset of symptoms. Second, we stratified our Day 1 analyses of IL-6 and D-dimer by presence or absence of severe sepsis to determine if an age-related immune response occurred among those who were most severely ill. Third, we repeated our analyses of IL-6 and D-dimer in subjects with similar etiology based on procalcitonin (PCT) stratification, since patients with high PCT (≥0.25 ng/ml) levels are more likely to have bacterial pneumonia [33]. PCT was measured using a time-resolved, amplified cryptate emission assay (Kryptor PCt, BRAHMS, Hennigsdorf, Germany) [34]. For sensitivity analyses, we analyzed three age groups: <65, 65–74, and ≥75 due to reduced sample sizes.

## Results

### Demographic and clinical characteristics

Table 1 shows the demographic and clinical characteristics for the 2183 subjects stratified by age group. Of the 2183 subjects, 495 (22.7%), 444 (20.3%), 403 (18.5%), 583 (26.7%), and 258 (11.8%) were <50, 51–64, 65–74, 75–84, and ≥85 years of age, respectively. The age range was 18–101 years, with a mean age of 67.2 years, and there were similar proportions of men and women in each age group. Older subjects had a higher burden of chronic disease compared to younger subjects, as evidenced by a higher Charlson score (p<0.001). Older adults were more likely to be in an assisted-living home and have restrictions on activities of daily living (ADL) (p<0.001). There was no age-related difference in the likelihood of receiving antibiotics in the week prior to ED arrival (p = 0.65). On average, older subjects experiencing symptoms presented to the ED 1–2 days earlier than younger subjects (p<0.001). Older subjects were more likely to have severe CAP upon presentation, as evidenced by greater proportion of older adults with a Pneumonia Severity Index score ≥IV and by a higher Acute Physiology and Chronic Health Evaluation (APACHE III) score (p<0.001 for both). Subjects stayed an average of 7 days in the hospital, with those ≥85 years staying on average 3 days longer than those <50 years.

**Table 1:** Demographic and clinical characteristics stratified by age.

Characteristic	<50 (n = 495)	50–64 (n = 444)	65–74 (n = 403)	75–84 (n = 583)	≥85 (n = 258)	Linear Trend P value
Demographic characteristics						
Sex male (%)	49.3	50.9	44.4	45.1	52.3	0.52
Race, white, n (%)	288 (58.2)	322 (72.5)	339 (84.1)	544 (93.3)	245 (95.0)	<0.001
Living Arrangement						
Home, n (%)	472 (95.4)	420 (94.8)	378 (93.8)	507 (87.1)	182 (70.5)	<0.001
Personal care home, unskilled assistance, n (%)	2 (0.4)	5 (1.1)	7 (1.7)	19 (3.3)	30 (11.6)	<0.001
Nursing care home, n (%)	1 (0.2)	10 (2.3)	16 (4.0)	51 (8.8)	43 (16.7)	<0.001
Other, n (%)	20 (4.0)	8 (1.8)	2 (0.5)	5 (0.9)	3 (1.2)	<0.001
Activities of Daily Living						
Fully independent and ambulatory, n (%)	445 (89.9)	338 (76.1)	271 (67.3)	312 (53.5)	74 (28.7)	<0.001
Mildly restricted, n (%)	45 (9.1)	89 (20.1)	103 (25.6)	199 (34.1)	104 (40.3)	<0.001
Housebound, n (%)	2 (0.4)	7 (1.6)	11 (2.7)	37 (6.4)	40 (15.5)	<0.001
Bed/chair bound, n (%)	3 (0.6)	10 (2.3)	18 (4.5)	35 (6.0)	40 (15.5)	<0.001
Comorbid conditions†						
Charlson score>0, n (%)	220 (44.4)	287 (64.6)	311 (77.2)	471 (80.8)	192 (74.4)	<0.001
Respiratory disease, n (%)	115 (23.2)	177 (39.9)	171 (42.4)	241 (41.3)	65 (25.2)	0.01
Renal disease, n (%)	15 (3.0)	16 (3.6)	11 (2.7)	8 (1.37)	1 (0.4)	0.003
Cardiac disease, n (%)	22 (4.9)	69 (17.7)	132 (37.7)	210 (41.4)	80 (33.9)	<0.001
Diabetes, n (%)	58 (11.7)	86 (19.4)	95 (23.6)	121 (20.8)	54 (20.9)	0.003
Neoplastic disease, n (%)	11 (2.2)	27 (6.1)	31 (7.7)	43 (7.4)	20 (7.8)	0.003
Ever smoked, n (%)	316 (64.1)	314 (71.4)	289 (72.4)	377 (65.1)	112 (44.3)	<0.001
Symptoms and therapy prior to hospitalization						
Duration of symptoms prior to hospitalization, mean (median, sd)	5.4 (3.0,8.1)	5.8 (3.0,8.1)	4.4 (3.0,6.4)	4.5 (3.0,6.8)	3.8 (2.0,4.9)	0.01
Received antibiotics within 7 days, n (%)	92 (18.6)	79 (17.8)	66 (16.4)	96 (16.5)	37 (14.5)	0.14
Severity of illness						
PSI class≥IV, n (%)	16 (3.2)	75 (16.9)	137 (34.0)	305 (52.3)	183(70.9)	<0.001
APACHE III, mean (median, sd)	35.8 (34, 19.2)	45.9 (45, 17.1)	56.9 (56, 15.5)	61.4 (59, 15.8)	68.1 (66, 15.3)	<0.001
Acute physiology score, mean (median, sd)‡	31.9 (32, 16)	36.7 (36, 15)	39.7 (38, 15)	41.4 (39, 14)	41.9 (40, 14)	0.30
Hospital length of stay, mean (median, sd)	4.8 (4, 5)	6.7 (5, 6)	6.7 (6, 5)	7.5 (6, 5)	7.7 (6, 6)	0.96

APACHE = Acute Physiology and Chronic Health Evaluation; PSI = Pneumonia Severity Index.

† Less than 2% of subjects reported pre-existing cirrhosis or AIDS.

‡ Acute physiology score derived from APACHE III score.

Blood or sputum cultures to determine microbiologic etiology were obtained in 1606 (74%) subjects within 48 hours of ED presentation. An etiologic agent was identified in only 186 (12%) of these subjects, with 110 (59%) identified as Gram-positive, 64 (35%) identified as Gram-negative, and 12 (6%) identified as mixed. Among subjects <65, 65–74, and ≥75, older subjects were more likely to have obtained a culture (65% vs. 78% vs. 81%) and similarly likely to have an etiologic agent identified (9% vs. 10% vs. 8%) (p<0.01 and p = 0.61, respectively). Older subjects tended to have a higher proportion of Gram-negative organisms compared to younger subjects (26% vs. 26% vs. 49% per increasing age group, respectively, with percentages totaling more than 100% because some subjects had more than one etiologic agent identified). A similar age-related trend was not seen in the proportion of Gram-positive organisms identified (50% vs. 18% vs. 30%).

### Older subjects have higher risk of severe sepsis and mortality

Severe sepsis occurred in a fourth of subjects (n = 588, Table 2). Compared to younger subjects, older subjects had a higher risk of severe sepsis (p<0.001), with those ≥85 years having a 2.8-fold increased risk of severe sepsis compared to those <50 years (42.3% vs. 14.8%, p<0.01). Older subjects were also more likely to develop respiratory, renal, neurological, and coagulation dysfunction while hospitalized (p≤0.02).

**Table 2:** Hospital outcome and mortality stratified by age.

Variable	<50	50–64	65–74	75–84	≥85	Linear Trend P value
Risk of severe sepsis and organ dysfunction						
Severe sepsis, n (%)	73 (14.8)	102 (23.0)	106 (26.3)	198 (34.0)	109 (42.3)	<0.001
Respiratory dysfunction, n (%)	45 (9.1)	72 (16.2)	69 (17.1)	106 (18.2)	42 (16.3)	0.001
Cardiac dysfunction, n (%)	11 (2.2)	23 (5.2)	9 (2.2)	27 (4.6)	5 (1.9)	0.76
Renal dysfunction, n (%)	30 (6.1)	47 (10.6)	53 (13.2)	124 (21.3)	77 (29.8)	<0.001
Liver dysfunction, n (%)	2 (0.4)	2 (0.5)	1 (0.3)	4 (0.7)	3 (1.2)	0.24
CNS dysfunction, n (%)	12 (2.4)	20 (4.5)	24 (6.0)	41 (7.0)	17 (6.6)	0.001
Coagulation dysfunction, n (%)	1 (0.2)	6 (1.4)	5 (1.2)	8 (1.4)	5 (1.9)	0.04
Mortality from ED admission						
Hospital mortality, n (%)	5 (1.0)	5 (1.1)	13 (3.2)	43 (7.4)	22 (8.5)	<0.001
90-day, n (%)	4 (0.8)	12 (2.7)	24 (6.2)	63 (11.7)	29 (12.3)	<0.001
Mortality after hospital discharge						
1-year, n (%)	17 (3.5)	35 (8.0)	62 (15.9)	130 (24.1)	75 (31.8)	<0.0001
Location after Discharge						
Home, n (%)	335 (91.3)	295 (84.8)	283 (76.3)	360 (64.0)	109 (44.1)	<0.0001
Acute or sub-acute care facility, n (%)	9 (2.5)	27 (7.8)	29 (7.8)	71 (12.6)	30 (12.2)	<0.0001
Assisted living facility, n (%)	6 (1.6)	15 (4.3)	38 (10.2)	83 (14.8)	82 (33.2)	<0.0001
Other, n (%)	17 (4.6)	11 (3.2)	21 (5.7)	48 (8.5)	26 (10.5)	0.06

ED = Emergency Department.

Older subjects had higher mortality during hospitalization and at 90 days after ED arrival (p<0.001 for all time points). Compared to subjects <50 years, those ≥85 years had an 8-fold increased hospital mortality (1.0% vs. 8.5%) and a 17-fold increased 90-day mortality (0.8% vs. 13.6%). Among survivors of CAP, there was also an age-related increase in mortality up to 1-year after discharge (p<0.001), with those ≥85 years having a 9-fold increase in mortality compared to those <50 years (31.8% vs. 3.5%) (Table 2).

### Differences in early immune response

**Figure 1:**
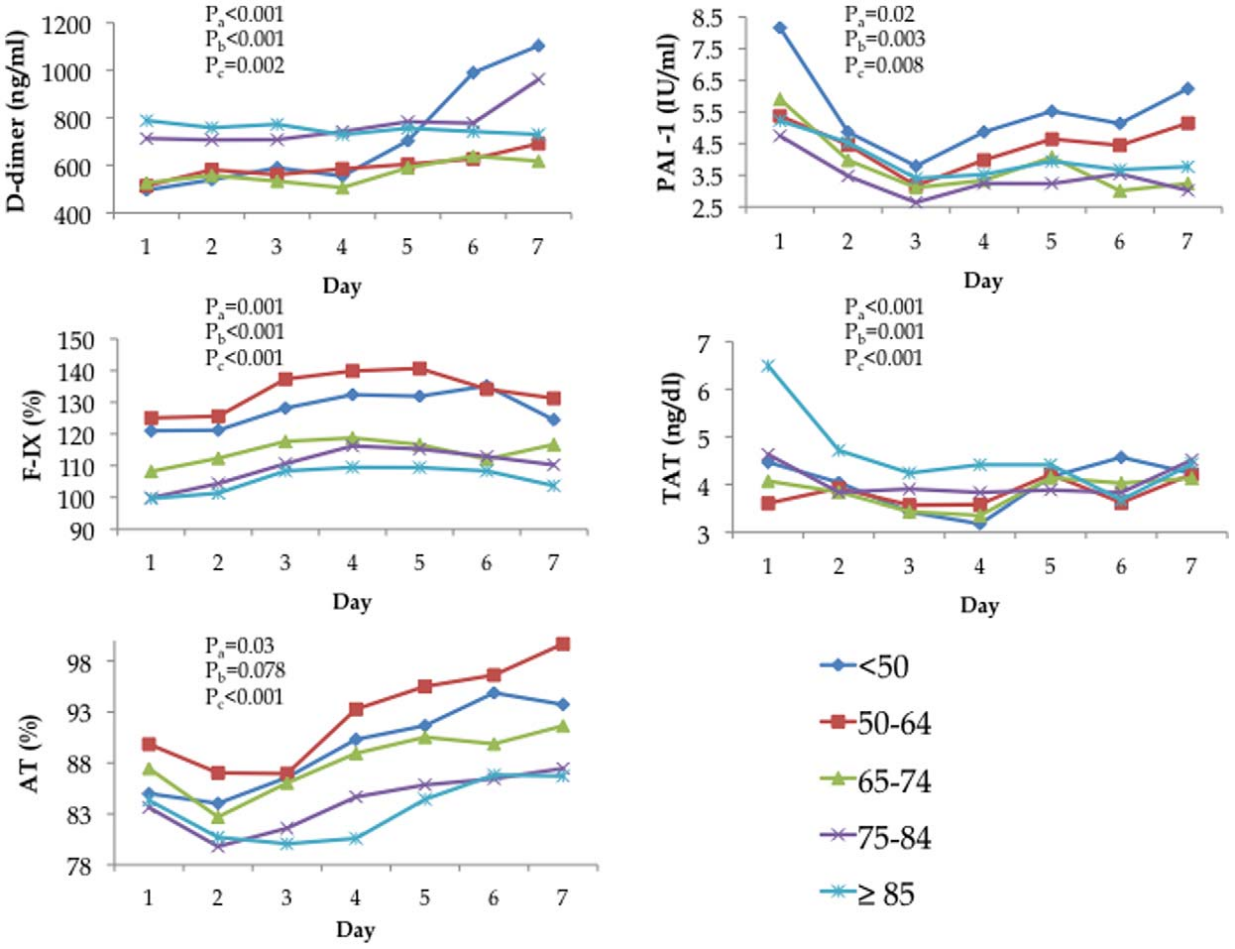
A modest pro-coagulant response was seen in older subjects upon ED admission (Day 1) and during the first week of hospitalization. There was an age-related increase in mean values of hemostasis markers D-dimer and thrombin-antithrombin complex (TAT) and an age-related decrease in F-IX, plasminogen-activator inhibitor-1 (PAI-1), and antithrombin (AT). Pa are p values from ANOVA or Tobit models adjusted for sex, race, comorbidity, and smoking status to compare differences in mean levels of markers on Day 1 across different age groups. Pb are p values for linear trend tests for markers on Day 1, adjusted for sex, race, comorbidity, and smoking status. Pc are p values from mixed effects model adjusted for sex, race, comorbidity, and smoking status to compare mean levels over the first week.

An age-related pro-coagulant response occurred in subjects upon arrival to the ED and continued throughout the first week of hospitalization. In multivariate analyses, subjects at ED presentation (Day 1) had age-related increases in circulating (mean) concentration of D-dimer (p<0.001) and TAT (p<0.001) and age-related decreases in PAI-1 (p = 0.02), AT (p = 0.03), and F-IX (p = 0.001) (Figure 1). With the exception of AT (linear trend p = 0.08), hemostasis markers showed a linear relationship with age (chi square for trend p<0.01 for all other markers, Figure 1). Numerically, these differences were modest: compared to those <50 years, subjects ≥85 years had 1.6-fold and 1.5-fold increases in D-dimer and TAT, respectively, and had 1.4-fold, 1.0-fold, and 1.2-fold decreases in PAI-1, AT, and F-IX, respectively. Mean values of AT increased over the first week, whereas the other four hemostasis markers had no clear trajectory. The age-related differences in mean concentration levels of all hemostasis markers at Day 1 persisted over the first week (all p<0.01, Figure 1).

**Figure 2:**
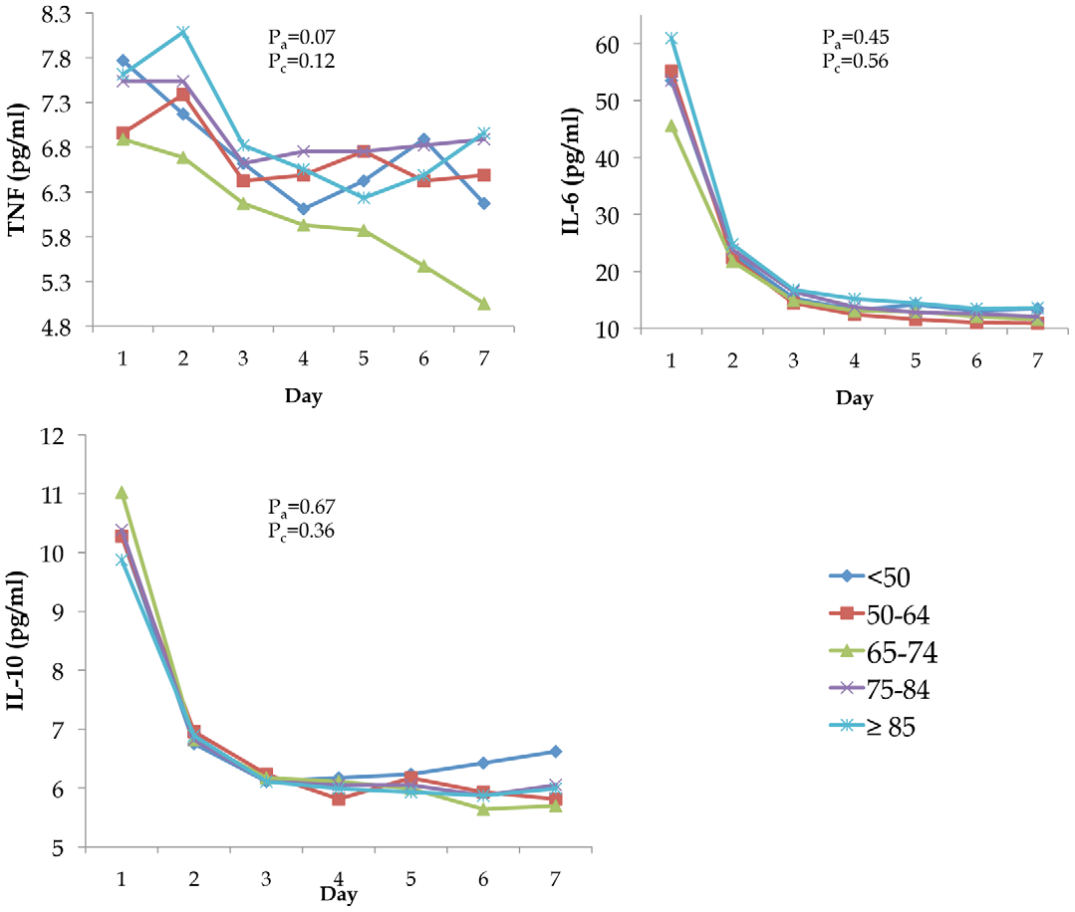
No differences were found in the mean values of inflammatory (TNF, IL-6, and IL-10) markers when stratified by age group. Markers were measured upon presentation to the ED and over the first week of hospitalization. Pa are p values from Tobit models adjusted for sex, race, comorbidity, and smoking status to compare differences in mean levels of markers on Day 1 across different age groups. Pc are p values from mixed effects model adjusted for sex, race, comorbidity, and smoking status to compare mean levels over the first week.

**Figure 3:**
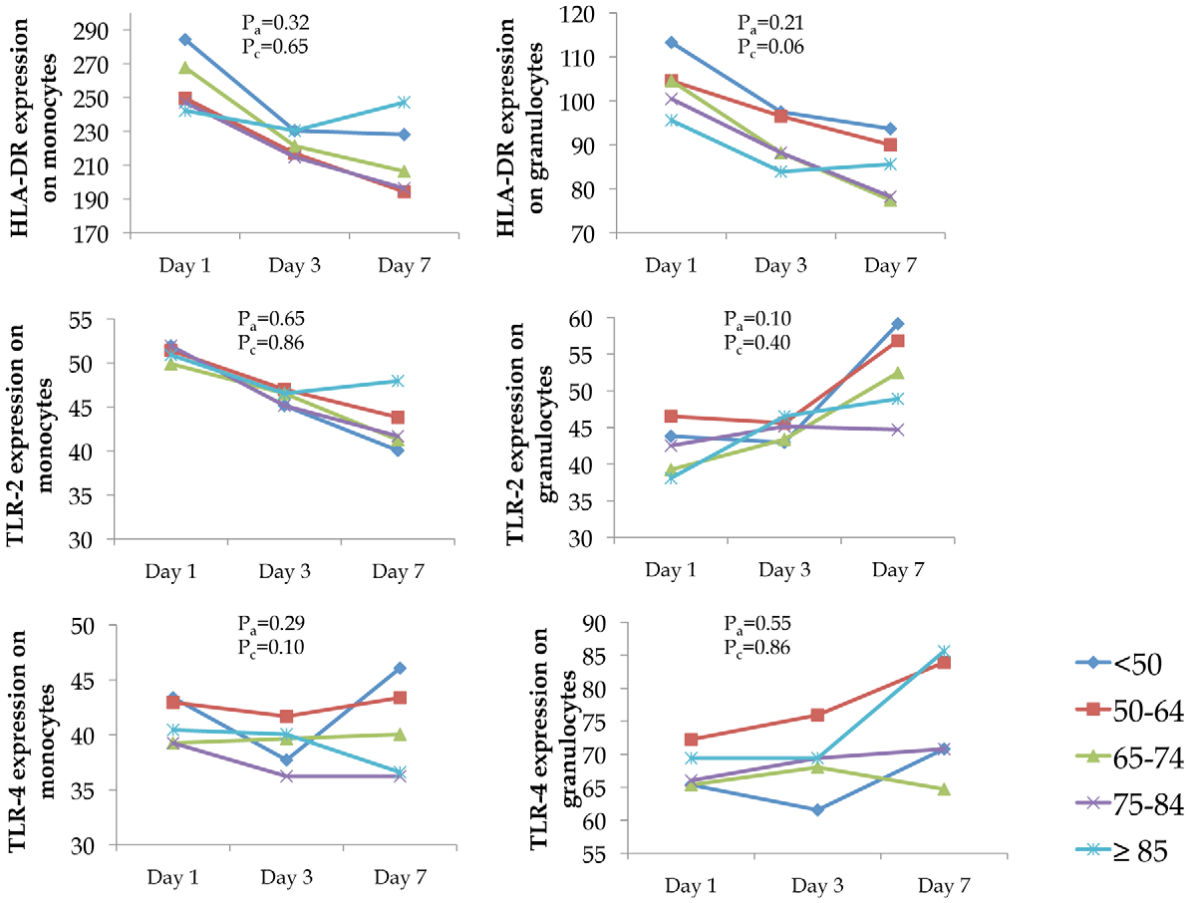
No differences were found in expression of cell surface markers when stratified by age group. Markers were measured upon ED presentation and at days 3 and 7. Values for cell surface markers are reported as mean channel fluorescence (MCF) of cells positive for a given cell surface marker. Pa are p values from ANOVA models adjusted for sex, race, comorbidity, and smoking status to compare differences in mean levels of markers on Day 1 across different age groups. Pc are p values from mixed effects model adjusted for sex, race, comorbidity, and smoking status to compare mean levels on Days 1, 3, and 7.

Circulating mean concentration levels of inflammatory markers IL-6, IL-10, and TNF and cell surface markers TLR-2, TLR-4, and HLA-DR were similar across different age groups on Day 1 and over the first week, respectively (Figures 2 and 3). Notable trajectories over the first week were IL-6 and IL-10, which both decreased sharply over the first three days. The frequency of IL-6:IL-10 groupings showed similar cytokine patterns across age groups (p = 0.32, Table 3). Over 70% of subjects in each age group were in either the low IL-6: low IL-10, medium IL-6: low IL-10, or medium IL-6: medium IL-10 category.

**Table 3:** Frequency (%) of group membership of IL-6:IL-10 patterns based on trajectory analysis over the first week of hospitalization.

	IL-6:IL-10 patterns								
Age	L∶L	L∶M	L∶H	M∶L	M∶M	M∶H	H∶L	H∶M	H∶H
<50	28.78	6.82	1.19	35.91	12.17	3.56	2.97	6.23	2.37
50–64	28.76	7.80	0.54	34.68	13.98	2.96	3.76	4.30	3.23
65–74	28.38	9.46	2.16	32.70	13.51	2.97	2.70	5.41	2.70
75–84	22.78	9.61	1.60	35.94	13.70	4.27	4.09	4.98	3.02
≥85	22.45	3.27	1.22	40.41	15.92	5.71	1.63	6.53	2.86

L = low; M = medium; H = high.

Distribution of IL-6:IL-10 sub-groupings did not differ across age group (P = 0.32).

Our analyses of hemostasis and inflammatory markers were not affected by time between the onset of symptoms of pneumonia and ED presentation, presence or absence of severe sepsis, or in subjects with PCT≥0.25. Among subjects who arrived to the ED within 24 hours of symptom onset, we found an age-related increase in mean levels D-dimer (p = 0.01) and no age-related difference in levels of IL-6 (p = 0.85). Likewise, in subjects with and without severe sepsis as well as in subjects with PCT≥0.25, there was an age-related increase in D-dimer (p = 0.02, <0.01, and 0.02, respectively) and no age-related difference in IL-6 (p = 0.86, 0.42, and 0.53, respectively) (Table 4).

**Table 4:** Day 1 mean and median levels of D-dimer (ng/ml) and IL-6 (pg/ml) stratified by severe sepsis, PCT, and those who arrived to ED within 24 hours of symptom onset.

Marker	<65	65–74	≥75	Linear Trend P value
Severe sepsis				
D-dimer, mean, median (n)	589.9, 658.5 (58)	749.9, 772.8 (41)	925.2, 1032.8 (135)	0.26
IL-6, mean, median (n)	76.7, 69.4 (116)	67.4, 79.8 (71)	83.1, 65.4 (239)	0.43
No severe sepsis				
D-dimer, mean, median (n)	502.7, 445.9 (168)	492.7, 454.9 (121)	645.5, 607.9 (201)	0.01
IL-6, mean, median (n)	49.4, 40.4 (388)	40.0, 33.8 (199)	43.8, 39.6 (397)	0.56
PCT≥0.25				
D-dimer, mean, median (n)	665.1, 678.6 (115)	685.4, 652.0 (75)	897.8, 1002.2 (146)	0.04
IL-6, mean, median (n)	145.5, 131.6 (241)	121.5, 107.8 (130)	139.8, 112.2 (275)	0.47
Arrival to ED within 24 hours of symptom onset				
D-dimer, mean, median (n)	391.5, 301.9 (53)	523.2, 572.5 (61)	796.3, 804.3 (128)	0.01
IL-6, mean, median (n)	75.9, 65.4 (116)	77.5, 66.7 (102)	80.6, 62.2 (244)	0.85

PCT = procalcitonin; ED = emergency department.

Table 5 shows ORs for the association of age group and 90-day mortality adjusted for pre-infection factors, such as demographics, chronic diseases, and smoking, and adjusted for pre-infection factors plus Day 1 hemostasis abnormalities. Compared to those less than 50 years, there was a higher risk of 90-day mortality in the 75–84 year (OR = 4.99; 95%CI = 1.46–17.13; p = 0.01) and ≥85-year (OR = 4.36; 95% CI = 1.19–15.97; p = 0.03) age groups when adjusted for baseline covariates. The ORs changed minimally when further adjusted for Day 1 hemostasis abnormalities in 75–84 year (OR = 4.79; 95% CI = 1.36–16.90; p = 0.01) or the ≥85-year (OR = 3.49; 95% CI = 0.93–13.11; p = 0.06) age groups.

**Table 5:** Unadjusted and adjusted odds ratios for association between age and mortality at 90 days.

	Odds Ratio (95% CI)					
Age	Unadjusted	P value	Adjusted†	P value	Adjusted with Day 1 hemostasis abnormalities‡	P value
<50	Referent		Referent		Referent	
50–64	0.96 (0.21–4.37)	0.95	0.71 (0.15–3.34)	0.67	0.81 (0.17–3.92)	0.80
65–74	2.66 (0.74–9.60)	0.13	1.86 (0.50–6.92)	0.36	2.15 (0.56–8.20)	0.26
75–84	7.42 (2.25–24.52)	0.01	4.99 (1.46–17.13)	0.01	4.79 (1.36–16.90)	0.01
≥85	6.20 (1.77–21.81)	0.01	4.36 (1.19–15.97)	0.03	3.49 (0.93–13.11)	0.06

CI = confidence interval.

† Adjusted for sex, race, comorbidity, and smoking status.

‡ Adjusted for sex, race, comorbidity, and smoking status, plus hemostasis abnormalities defined as follows: D-dimer >256 ng/ml, PAI-1 activity >31 IU/ml, Antithrombin activity <70%, Factor IX activity <60%, and TAT >5.0 ng/ml.

### Differences in immune resolution at hospital discharge

Across all age groups, most subjects were discharged to home (Table 2). However, older adults were more likely to be transferred to an acute or sub-acute care facility or a nursing home upon discharge. Most subjects met the Halm criteria for clinical stability on the day of discharge. Only 15 (2%), 73 (8%), 24 (3%), and 8 (1%) subjects had a temperature higher than 37.8°C, heart rate higher than 100/minute, respiratory rate higher than 24/minute, or systolic blood pressure <90 mm of Hg, respectively. Older adults were more likely to have all 4 vital signs returned to normal at discharge (81% vs. 85% vs. 87% vs. 90% vs. 90%) (p<0.01) and were equally likely to be discharged with 2 or more abnormal vital signs (3% vs. 3% vs. 2% vs. 1% vs. 1%) (p = 0.3).

An age-related pro-coagulant response was observed in survivors upon hospital discharge (Table 6). In multivariate analysis, older subjects had significantly increased levels of D-dimer (p<0.001) and TAT (p = 0.01) and significantly decreased levels of PAI-1 (p = 0.002), AT (p<0.001), F-IX (p<0.001). All coagulation markers showed a linear relationship with age (p<0.01). Compared to those <50 years, subjects ≥85 years had 1.2-fold and 1.4-fold increases in D-dimer and TAT, respectively, and 1.3-fold, 1.1-fold, and 1.2-fold decreases in PAI-1, AT, and F-IX, respectively.

**Table 6:** Mean values of inflammation and hemostasis markers prior to discharge from hospital show a modest age-related increase in inflammation and coagulant response.

Markers	<50yrs	50–64yrs	65–74yrs	75–84yrs	≥85yrs	ANOVA P value†	Linear Trend P value‡
Inflammation (pg/ml)							
TNF	6.6	6.2	6.4	6.5	6.3	0.27	0.10
IL-6	9.8	10.1	10.8	10.8	11.8	0.02	<0.001
IL-10	6.0	5.8	5.9	5.8	5.9	0.83	0.69
Hemostasis							
D-dimer (ng/ml)	607.9	550.0	518.0	727.8	699.2	<0.001	0.009
PAI-1 (IU/ml)	5.8	5.5	4.7	4.0	4.3	0.002	0.002
F-IX (%)	126.5	134.3	119.1	107.8	106.7	<0.001	<0.001
TAT (ng/ml)	3.2	3.3	3.7	3.8	4.5	0.01	<0.001
AT (%)	97.5	96.5	91.8	88.2	85.6	<0.001	<0.001

F-IX = Factor IX; PAI-1 = plasminogen-activator inhibitor-1; TAT = thrombin-antithrombin complex; AT = antithrombin.

† P values from ANOVA or Tobit models adjusted for sex, race, comorbidity, and smoking status.

‡ P values from linear trend test adjusted for sex, race, comorbidity, and smoking status.

Despite similar levels of inflammatory markers at ED presentation, subjects at hospital discharge showed an age-related increase in IL-6 (p = 0.02), and this relationship was linearly related with age (p<0.001). Compared to those <50 years, subjects ≥85 years had a 1.2-fold increase in IL-6. Mean concentrations of inflammatory markers IL-10 and TNF were similar across age groups at discharge.

## Discussion

Surprisingly, unlike the large age-related differences in circulating inflammatory and coagulation-fibrinolysis response seen in animal models of infection, we did not observe differences in circulating inflammatory response and showed only modest differences coagulation-fibrinolysis response between older and younger subjects hospitalized for CAP. The modest differences in hemostasis markers did not explain the large age-related differences in mortality. Our results remained unchanged when repeated to account for differences in time between the onset of symptoms and hospitalization, and in subgroups with and without severe sepsis. Furthermore, we did not observe differences in anti-inflammatory response, as evidenced by similar levels of IL-10 and in IL-6:IL-10 ratios across different age groups. We did show age-related differences in immune resolution among survivors of CAP, with older subjects having a pro-inflammatory and pro-coagulant response compared to younger subjects at hospital discharge. These findings have important implications for understanding age-related differences in long-term outcomes of sepsis.

Large-related differences in 90-day mortality occurred among our subjects despite a similar circulating immune response to pneumonia during hospitalization. There are several explanations for this occurrence, including potential immune response differences that occurred prior to hospitalization and possible differences that occurred at the tissue level and were not measured in our study. However, we speculate that the large differences in early outcome in our subjects are likely driven by underlying frailty in the older population, which is characterized by vulnerability to acute stressors and is a consequence of decline in overall function and physiologic reserve [35]. In the U.S., nearly 7% of those over 65 and 30% over 80 years are considered frail [36]. In our cohort, over 30% of subjects ≥85 reported significant impairment in activities of daily living, which is a crude marker for frailty [37]. Our results show that older subjects had increased organ dysfunction and severe sepsis despite small differences in circulating inflammatory and coagulation-fibrinolysis response, suggesting that older adults may have increased susceptibility to end-organ damage by inflammatory and coagulant mediators than younger adults.

Large age-related differences in mortality persisted among survivors of CAP hospitalization at 1-year after hospital discharge. Prior studies and our results suggest that the higher mortality after hospital discharge among older adults is not explained by higher burden of chronic diseases prior to infection [38]. We found a modest age-related increase in inflammatory and hemostasis markers at discharge, despite apparent clinically recovery from pneumonia. Although we could not measure pre-infection inflammatory and hemostasis markers in our subjects, the marker levels found at discharge are at least 4-fold higher than have been reported at baseline in community-dwelling individuals with similar ages, suggesting an unresolved immune response in our subjects [38], [39], [40]. We speculate that a persistent pro-inflammatory and pro-coagulant state during recovery among older adults may explain higher risk of long-term sequelae among the elderly [41], [42], [43].

Prior studies have shown increased PAI-1 levels in older adults and among those with higher illness severity following an infection [15], [44], [45]. Although older subjects were more severely ill, we observed lower circulating PAI-1 levels among older subjects in our study. Potential reasons for lower circulating PAI-1 concentrations in the elderly include downregulation of PAI-1 expression due to sustained IL-10 expression and age-related changes in tensile strength of tissues and cells, which have been shown to decrease PAI-1 expression [46], [47]. Our findings are consistent with a recent study of elderly patients with periodontal disease, where PAI-1 was unique among hemostasis markers in being lower among those with severe periodontal disease [48].

Our study has three major strengths. First, unlike similar cross-sectional studies [18], [49], [50], we measured immune markers on ED presentation and during hospital course, and were thus able to compare both the initial immune response in patients at the time of ED presentation and its subsequent trajectory. In most subjects, samples were obtained in the ED prior to initiation of antibiotic therapy and other interventions. Thus our results are less likely to be confounded by therapeutic interventions. Second, we analyzed a large multicenter cohort of subjects with a single infectious disease, CAP. We chose CAP because it is the most common infectious cause of hospitalization in developed countries and the most common cause of severe sepsis [1], [51]. Our results are thus generalizable and less likely to be confounded by differences in source of infection. Third, we measured expression of TLR-2, TLR-4, and HLA-DR because these receptors are integral components of the host response to infection and are involved in activating the inflammatory and coagulant response to infection [52], [53], [54]. Our finding that all three cell surface markers remained similar across age groups corroborates and strengthens our analysis of little or no differences in inflammatory or coagulation-fibrinolysis response.

Our findings have limitations. First, due to practical reasons we did not assess the immune response prior to hospitalization. Larger differences in immune response may occur during this period and likely lead to higher risk of hospitalization among older adults. Second, we could not fully assess whether our results were confounded due to potential age-related differences in infectious etiology. We obtained a low yield of bacterial cultures in our subjects, which is consistent with previous large studies of CAP patients and is likely due to poor yield of current culture techniques [55]. We therefore repeated key analyses stratified by high PCT level to compare subjects who are more likely to have bacterial infection, and showed that inflammatory and coagulation marker patterns were similar in this subset.

In conclusion, our results suggest that, once hospitalized for CAP, there is little age-related difference in circulating immune response. The differences in coagulation-fibrinolysis response that exist do not explain the large differences in outcome observed between younger and older adults. Finally, older patients who survive CAP may have delayed immune resolution compared to younger patients.
